# Transcriptomic and Root Microbiome Responses of Lettuce to Beneficial Endophytic Bacteria in Hydroponic Systems

**DOI:** 10.3390/ijms27073072

**Published:** 2026-03-27

**Authors:** Bimal Sajeewa Amaradasa, Robert L. Chretien, Scott Lowman, Chuansheng Mei

**Affiliations:** Plant Endophyte Research Center, Institute for Advanced Learning and Research, Danville, VA 24540, USA; sajeewa.amaradasa@ialr.org (B.S.A.);

**Keywords:** *Pseudomonas psychrotolerans*, bacterial endophytes, transcriptome, root microbiome, hydroponic system, *Lactuca sativa*

## Abstract

Controlled environment agriculture (CEA) relies on hydroponic systems to achieve high yields, yet optimizing plant performance remains a challenge. Beneficial endophytic bacteria offer a sustainable solution by promoting growth and nutrient uptake. Here, we investigated the mechanistic basis of growth enhancement in lettuce (*Lactuca sativa*) inoculated with *Pseudomonas psychrotolerans* IALR632 in a nutrient film technique (NFT) system. Growth measurements showed significant increases in shoot and root biomass and leaf greenness. RNA-seq profiling at 4, 10, and 15 days after transplanting revealed dynamic transcriptional reprogramming, with 38, 796, and 7642 differentially expressed genes, respectively. MapMan and GO analyses indicated up-regulation of pathways related to cell wall remodeling, lipid metabolism, nitrogen assimilation, and stress adaptation, alongside modulation of ethylene signaling. Root bacterial microbiome through 16S metabarcoding sequencing demonstrated distinct community shifts, confirmed by analysis of similarity (ANOSIM) (R = 1, *p* = 0.028), with enrichment of genera linked to nutrient cycling and plant growth promotion. These findings provide integrated molecular and ecological evidence that IALR632 enhances lettuce growth by coordinating host gene expression and rhizobiome restructuring, offering a mechanistic framework for microbial inoculant strategies in hydroponic horticulture.

## 1. Introduction

Controlled environment agriculture (CEA) is increasingly essential for ensuring global food security, as urbanization accelerates and arable land becomes limited. Leafy green vegetables, particularly lettuce, are ideal candidates for CEA due to their rapid growth, high yield, and superior produce quality. However, the widespread adoption of hydroponic systems—a key component of CEA—is challenged by high initial infrastructure costs, which can be offset by increased crop productivity.

Plant growth-promoting bacteria (PGPB) have gained significant attention in sustainable agriculture for their ability to enhance plant growth, improve nutrient uptake, increase stress tolerance, and suppress pathogens. In hydroponic systems, PGPB have been shown to improve plant performance and contribute to overall crop quality [1,2,3,4]. Several studies have demonstrated the beneficial effects of PGPB in hydroponic lettuce production [1,5,6,7,8]. Among these, *Pseudomonas* spp. represents a diverse group of Gram-negative, rod-shaped, motile Υ-proteobacteria, known for their plant-beneficial traits, including growth promotion, abiotic stress tolerance, and pathogen inhibition [8,9,10,11]. For instance, *Pseudomonas* sp. LSW25R enhanced tomato growth, increased calcium uptake, and reduced disease incidence in hydroponic systems [11]. Similarly, *Pseudomonas chlororaphis* treatment improved biomass and height in romaine lettuce grown in a window hydroponic setup [12]. These bacteria promote nutrient uptake, produce phytohormones, enhance microbial diversity, and reduce reliance on chemical inputs, offering a sustainable approach to crop management. PGPB enhances lettuce growth in hydroponic systems through multiple mechanisms: solubilizing nutrients, fixing atmospheric nitrogen, and producing growth regulators such as auxins and vitamins. They also mitigate abiotic stresses by lowering ethylene levels via ACC deaminase activity and protect plants against pathogens through antibiotic production and competitive exclusion [13].

Many PGPB act as endophytes, entering plants through root hairs, lateral root emergence sites, germinating radicles, secondary roots, or occasionally through stomata [14]. Entry is aided by cell wall-degrading enzymes such as cellulases and pectinases [15]. After penetration, endophytes colonize internal tissues, including roots, stems, leaves, seeds, and vascular tissues—most often in intercellular spaces. Their populations can reach 10^3^–10^5^ CFU per gram of tissue, depending on plant organ and environmental conditions. Our previous research showed that IALR632 had a population at 1.01 × 10^5^ and 3.87 × 10^4^ CFU/g tissues in roots and leaves, respectively [16]. Once inside, endophytes engage in active metabolic exchange with their host. They utilize plant-derived carbohydrates, amino acids, and organic acids while producing osmoprotectants, antioxidants, and phytohormones that enhance plant growth and stress tolerance. These interactions extend to molecular signaling, influencing plant gene expression and contributing to improved physiological performance [17].

A healthy root microbiome is essential for optimal plant health and development. However, little is known about how PGPB influence root-associated microbial communities and host gene expression in hydroponic environments. Recent hydroponic lettuce studies show that PGPB inoculation can restructure root microbiomes and enhance plant defense responses, and pathogen pressures can further shift microbial assemblages in soilless systems. Microbial colonization is also known to induce extensive transcriptional reprogramming in plants, including modulation of immune receptor families and hormone-regulated transcription factors that shape root exudation and microbiome assembly. Although Lee et al. (2016) reported transcriptomic and microbial shifts in lettuce inoculated with beneficial bacteria [12], the mechanisms underlying these interactions in hydroponic systems remain poorly defined [18,19,20].

To address this gap, we investigated the effects of *Pseudomonas psychrotolerans* IALR632 on lettuce grown in an NFT hydroponic system, examining plant gene expression (RNA-seq) and root bacterial microbiome composition (16S metabarcoding). We hypothesized that IALR632 inoculation simultaneously restructures the root microbiome and modulates host transcriptional pathways, thereby driving plant growth promotion in hydroponic conditions.

## 2. Results

### 2.1. Lettuce Growth Promotion by IALR632

**Figure 1:**
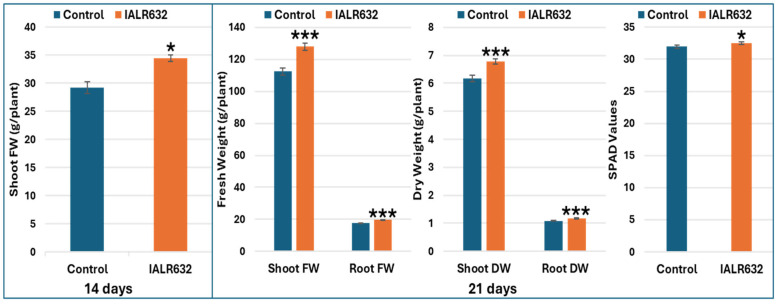
IALR632 promoted lettuce plant growth in NFT units. Asterisks (*, ***) indicate significant differences from the control at *p* < 0.05 and *p* < 0.001, respectively.

Inoculation with IALR632 significantly enhanced lettuce growth in NFT units (Figure 1). Shoot fresh weight increased by 18.2% on day 14 and 13.9% on day 21 relative to the control. On day 21, root fresh weight, root dry weight, and leaf greenness (SPAD value) were also significantly higher in IALR632-treated plants.

### 2.2. Lettuce Transcriptome Analyses

Approximately 90% of RNA-seq reads of each sample aligned to the reference genome. Of aligned reads, approximately 76–80% were assigned to annotated features of the reference genome. The average transcriptome size of samples in each treatment, as well as the aligned and assigned percentages of reads to the reference genome, is documented in Supplementary Table S1.

The reference genome cv. Salinas v11 had 70,163 transcript IDs. They belonged to 48,985 unique genes. There were 1115 unassigned transcript IDs. The corresponding gene IDs of differentially expressed transcripts (*padj* < 0.01) at 4, 10, or 15 days after transplanting (DAT) were all unique, indicating that each transcript ID mapped to a distinct gene ID. The set of reference gene lists (background genes) filtered for DAVID analysis contained 24,322, 24,345, and 24,620 genes for comparisons conducted at 4, 10, and 15 DAT, respectively.

**Figure 2:**
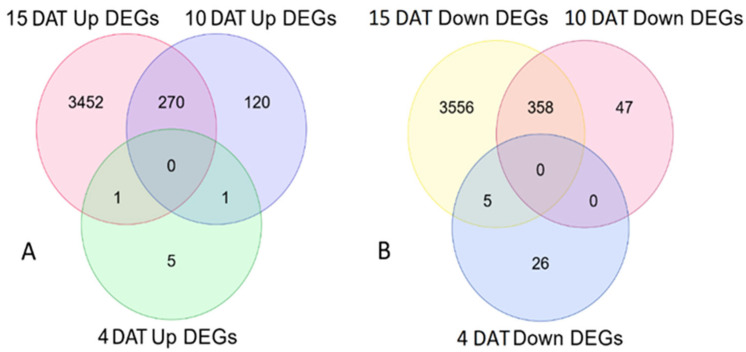
Venn diagrams of differentially expressed genes (DEGs) (*padj* < 0.01) for lettuce (*Lactuca sativa* cv. Green Oakleaf). Plants had been inoculated with IALR632 and compared with non-inoculated control for gene expression at 4, 10, and 15 days after transplanting (DAT). (**A**): Unique and common up-regulated genes; (**B**): Number of down-regulated genes at 4, 10, and 15 DAT.

Separate Venn diagrams for up- and down-regulated DEGs for the three sampling points are given in Figure 2A and Figure 2B, respectively. There were no up- or down-regulated genes common to all three comparisons. More genes were differentially expressed in plants sampled at 15 DAT compared to the other two comparisons. Plants sampled at 4 DAT had a total of 38 genes (both up- and down-regulated), while the plants at 10 and 15 DAT resulted in 796 and 7642 DEGs, respectively.

The DESeq2 differential expression results and transcript-level metadata for lettuce (*Lactuca sativa* cv. Green Oakleaf) transcriptomes were analyzed at 4, 10, and 15 DAT and are listed in Supplementary Table S2. The table integrated normalized count data generated by DESeq2 from transcriptomic read counts produced via the read mapping and transcript assembly (RMTA) pipeline and included corresponding gene annotations from the *L. sativa* cv. Salinas v11 reference genome.

Volcano plots were generated to facilitate the visualization of up- and down-regulated genes at 10 and 15 DAT. At 10 DAT, 210 genes met the criteria of |log2 fold change| > 1 and adjusted *p* value < 0.01. At 15 DAT, the number of genes satisfying these thresholds increased markedly to 2342, representing a substantial rise in differential expression (Supplementary Figure S1).

### 2.3. Metabolic Pathway Changes

**Figure 3:**
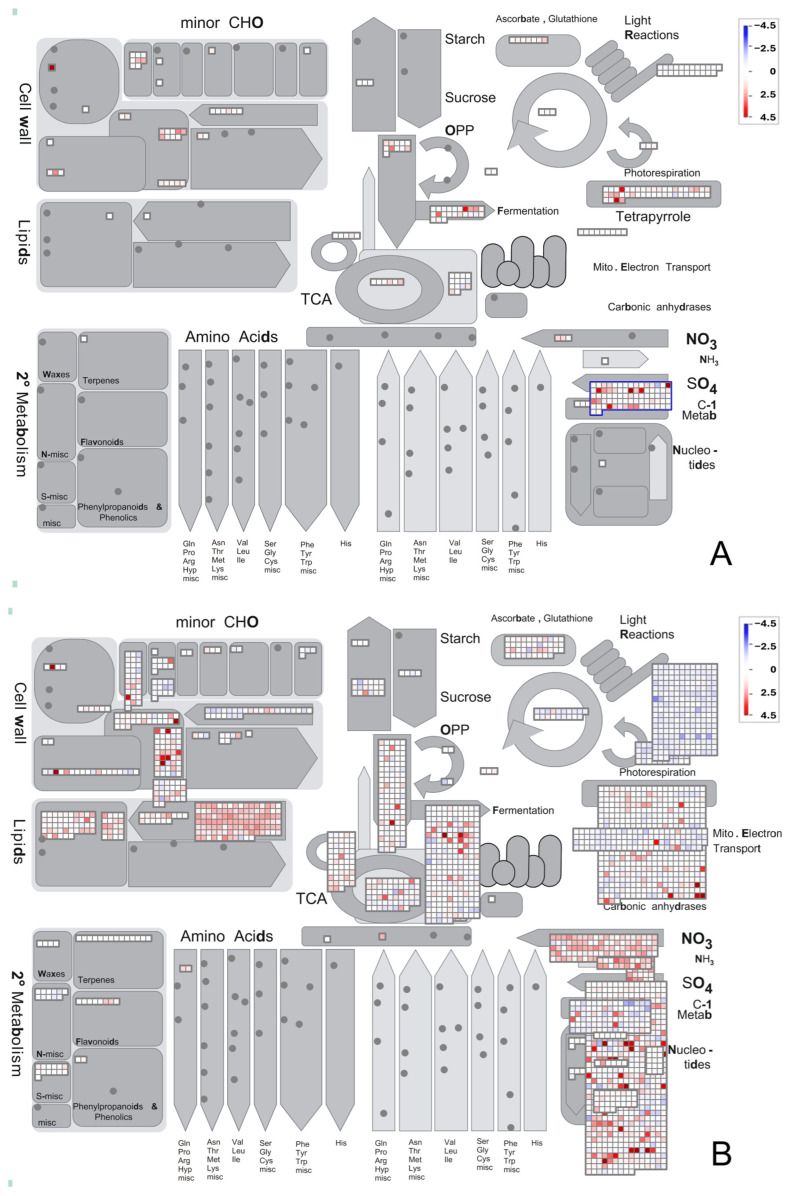
Overview of metabolic changes in lettuce leaves at 10 days (**A**) and 15 days (**B**) after transplanting, visualized with MapMan. Genes significantly up-regulated in bacteria-treated leaves are shown as red squares, while down-regulated genes are shown as blue squares. Scale bars display log2-fold changes, and only significant differences are displayed. OPP: oxidative pentose phosphate pathway; TCA: tricarboxylic acid cycle.

MapMan was used to show gene expression changes in the metabolic pathways. At 10 DAT, it showed that some genes were up-regulated in cell wall, tricarboxylic acid cycle (TCA), oxidative pentose phosphate (OPP), fermentation, sulphate, and tetrapyrrole pathways (Figure 3A). These are related to plant growth and related anabolic activities. Up-regulation of some transcripts in fermentation pathway may be an adaptive stress response mechanism to low oxygen levels in the root zone of hydroponic plants. More genes related to sulphate pathway were also up-regulated, which indicate active nutrient assimilation and the synthesis of essential sulfur-containing compounds vital for growth and survival [21,22]. The tetrapyrrole biosynthesis pathway is a fundamental metabolic route producing vital molecules like chlorophyll (photosynthesis), heme (respiration, oxygen transport), siroheme (sulfur/nitrogen assimilation), and vitamin B12 [23,24].

At 15 DAT, large numbers of genes were up-regulated (Figure 3B). Up-regulation of cell wall and lipid pathways indicated active remodeling and strengthening of cell walls supporting faster or sturdier development. Lipid metabolism suggests enhanced synthesis of membrane and storage lipids (Figure 3B). Lipids are crucial for cell division, signaling, and stress adaptation [24]. Their up-regulation points to active growth and possibly improved membrane integrity under bacterial inoculation. Nitrate (NO_3_^−^) and ammonia (NH_3_) metabolisms had also increased, reflecting improved nitrogen uptake and assimilation. However, light reactions (photosynthesis) showed down-regulation, which suggests reduced activity of photosynthetic electron transport. This could mean the plant is reallocating energy away from photosynthesis toward growth and nutrient assimilation, or that bacterial treatment alters chloroplast activity at this stage. Down-regulation of photorespiration is also visible on the MapMan plot (Figure 3B). This is generally beneficial as less energy is wasted on oxygen fixation. This implies improved carbon efficiency, potentially allowing more resources to be directed toward biomass and metabolite production.

### 2.4. Biological Process

**Figure 4:**
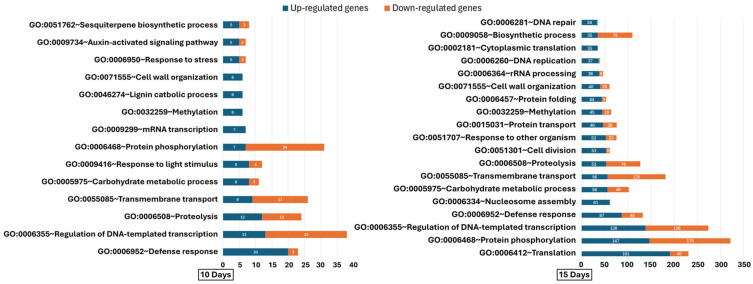
Differential expressions of top up-regulated Gene Ontology (GO) Biological Process (BP_Direct) terms in lettuce (*Lactuca sativa* cv. Green Oakleaf) at 10 days and 15 days after transplanting. The stacked bar chart shows the number of up-regulated genes (blue) annotated to each BP_Direct term, along with the corresponding down-regulated genes (orange) associated with the same terms. GO terms were selected based on annotation frequency, not statistical enrichment. Gene expression was assessed relative to non-inoculated control plants, highlighting biological processes influenced by inoculation with the growth-promoting bacterial strain IALR632.

Gene Ontology (GO) Biological Process (BP) terms annotated by DAVID (GOTERM_BP_DIRECT) were analyzed at 4, 10, and 15 DAT to track temporal changes in gene functions. These terms provide broad, hierarchical functional annotations. At 4 DAT, no enriched GO terms were found for up-regulated genes; six down-regulated terms were identified (Supplementary Table S3), with cellular oxidant detoxification (GO:0098869) and protein folding (GO:0006457) being most significant. At 10 DAT, 222 up-regulated and 235 down-regulated genes were linked to BP terms. Defense response (GO:0006952) had 20 up-regulated genes, versus three down-regulated. Other key processes showing mixed regulation included DNA-templated transcription, proteolysis, and transmembrane transport (Figure 4). Growth-related processes such as mRNA transcription, lignin catabolism, response to light, and response to stress were enriched among up-regulated genes (Supplementary Table S4), indicating early activation of developmental pathways. By 15 DAT, transcriptional changes intensified: 2351 up-regulated (63.1%) and 2200 down-regulated (56.1%) genes were associated with BP terms. The up-regulated genes were mainly involved in growth and proliferation—translation (191 genes), protein phosphorylation (147 genes), and DNA-templated transcription regulation (138 genes). Down-regulated genes also clustered in protein phosphorylation and transcriptional regulation, suggesting a complex cellular rebalancing (Figure 4). Enriched up-regulated terms such as translation, DNA replication, cell division, and ribosome biogenesis support enhanced development, while down-regulated terms indicate reduced investment in light harvesting and stress response (Figure 4, Supplementary Table S5).

The GO terms consistently observed at high frequency at both 10 and 15 DAT were examined to elucidate the sustained physiological reprogramming of the lettuce host induced by the endophyte IALR 632 (Supplementary Table S6). Up-regulated DEGs included auxin-activated signaling (GO:0009734) and cell wall organization (GO:0071555), which are linked to the amplification of the plant’s internal growth pathways. Additionally, there was a high frequency of genes involved in carbohydrate metabolic processes (GO:0005975), defense response (GO:0006952), and methylation related regulatory and signaling processes (GO:0032259) among the up-regulated genes. The most frequent down-regulated processes included fatty acid biosynthetic processes (GO:0006633). Notably, several categories appeared in both the up- and down-regulated datasets at high frequencies, specifically the regulation of DNA-templated transcription (GO:0006355), protein phosphorylation (GO:0006468), transmembrane transport (GO:0055085), and proteolysis (GO:0006508).

### 2.5. Growth and Stress-Related Genes

Given the higher biomass in bacterium-treated plants, DEGs at 10 and 15 DAT were examined for growth and stress-related genes. The acid phosphatase (AP) gene LOC111898238 was consistently up-regulated at both time points. At 10 DAT, purple acid phosphatase (PAP) gene LOC111904408 also exhibited increased expression, with no phosphate-related genes down-regulated. By 15 DAT, additional PAP (LOC111918260, LOC111915916) and AP (LOC111898238, LOC111904674) genes were up-regulated, indicating sustained phosphate mobilization, though three PAP genes were down-regulated (Supplementary Table S7).

Endophytic bacteria producing ACC deaminase can lower ethylene levels by degrading ACC, potentially reducing ethylene-responsive gene expression [25,26]. Thus, ethylene-responsive transcription factors (ERFs) and ethylene biosynthesis genes (ACS, ACO) were examined. At 10 DAT, three ACO genes were up-regulated and four down-regulated. At 15 DAT, eight ACO genes were down-regulated versus four up-regulated (Supplementary Table S6). No ACS genes were differentially expressed, and ERFs showed no significant down-regulation for either sampling dates.

At 15 DAT, enriched BP terms indicated down-regulation of hormone response pathways for ethylene (GO:0009723), auxin (GO:0009733), gibberellin (GO:0009739), and abscisic acid (GO:0009737), while other hormone-related processes and stress responses—such as response to water deprivation O:0009414) and cellular response to heat (GO:0034605)—were up-regulated (Supplementary Table S5).

### 2.6. Root Bacterial Microbiome Analysis

#### 2.6.1. Sequencing Data Quality and Filtering

After sequencing data quality check, the distribution of effective sequence lengths is shown in Figure S2. The rarefaction curves reached saturation, indicating sufficient sequencing depth for comprehensive characterization of bacterial diversity across samples (Figure S3).

#### 2.6.2. Operational Taxonomic Unit (OTU) Clustering and Abundance

A total of 653 OTUs were identified across all samples. Of these, 574 OTUs were shared between the control and IALR632-inoculated groups, while 52 were unique to the inoculated samples, and 27 were unique to the controls. A heatmap of the 30 most abundant OTUs revealed both similarities and differences between the control and treatment groups (Figure S4).

#### 2.6.3. Bacterial Community Diversity

**Figure 5:**
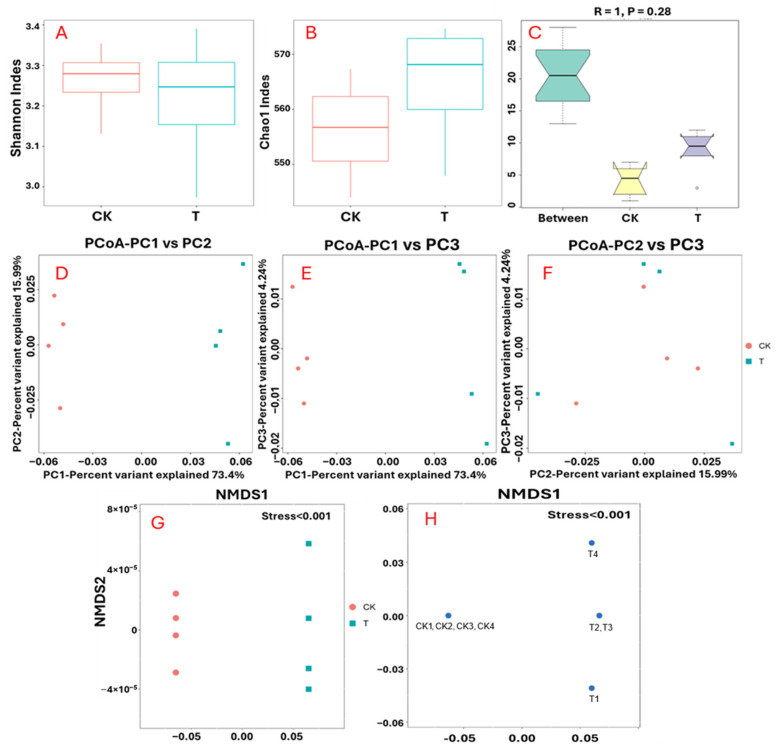
Changes of bacterial community diversities after bacterial inoculation. (**A**): Shannon indices; (**B**): Chao1 indices; (**C**): ANOSIM analysis; (**D**–**F**): PCoA analyses; (**G**,**H**): NMDS analysis.

Alpha diversity: alpha diversity was assessed using Chao1 and Shannon indices to evaluate richness and evenness within each group. The Shannon index indicated higher microbial diversity in IALR632-treated samples compared to controls (Figure 5A), while Chao1 values also reflected increased richness in the treated group (Figure 5B).

Beta diversity: beta diversity analysis was performed to assess differences in microbial community composition between groups. ANOSIM analysis: to statistically validate differences in community composition, analysis of similarity (ANOSIM) was performed. The analysis yielded an R-value of 1 and a *p*-value of 0.028, indicating that between-group differences (treatment vs. control) were significantly greater than within-group variation, confirming a strong treatment effect on the root microbiome (Figure 5C). Principal Coordinates Analysis (PCoA) based on Bray–Curtis dissimilarity revealed clear separation between IALR632-treated and control samples, suggesting distinct community structures (Figure 5D–F). NMDS analysis: Non-metric multidimensional scaling (NMDS) positions each object in multidimensional space based on functional classification information, with distances between points representing their dissimilarities. The resulting spatial plot reflects these relationships (Figure 5G,H). Stress values were <0.001, indicating an excellent fit (values from 0.001 to ≤0.05 are considered a good fit, and values > 0.05 to ≤0.1 are considered fair), which indicated that the bacteria-treated group was clearly separated from the control group based on their functional classification information.

#### 2.6.4. Taxonomic Composition at Phylum and Genus Levels

**Figure 6:**
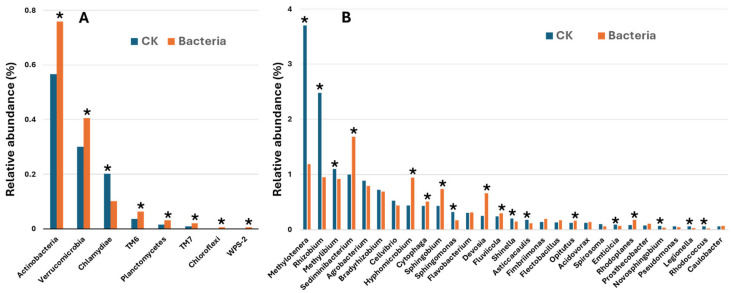
Relative abundance of Phyla and genera in the bacteria-treated group and the control group. (**A**): phylum relative abundance. (**B**): genus relative abundance. Asterisks (*) indicate significant differences from the control at *p* < 0.05.

At the phylum level, eight out of the 18 detected phyla showed significant differences in relative abundance between IALR632-treated and control samples (Figure 6A). Of these, only Chlamydiae was significantly reduced in population (−50%) in the treated group compared with the control. The remaining seven phyla were significantly enriched, with increases ranging from +34% to +224% relative to controls.

At the genus level, a total of 83 genera were identified across all samples. The dominant taxon in both treatments was an unclassified genus, representing 85.04% in controls and 86.93% in IALR632-treated samples (*p* = 0.0135). Relative abundance patterns for the 30 most abundant genera are shown in Figure 6B. Among the top genera, bacterial inoculation markedly reduced the relative abundance of *Methylotenera* (−68%), *Rhizobium* (−62%), *Methylibium* (−16%), and *Sphingomonas* (−45%). In contrast, it substantially increased the abundance of *Sediminibacterium* (+68%), *Hyphomicrobium* (+114%), *Sphingobium* (+72%), *Devosia* (+158%), *Mycobacterium* (+513%), *Rhodoplanes* (+103%), and *Runella* (+1920%).

## 3. Discussion

The plant endophyte *Pseudomonas psychrotolerans* IALR632 is a growth-promoting bacterium primarily known for its ability to solubilize insoluble phosphates, along with siderophore production and ACC deaminase activity [27]. Previous studies have demonstrated significant growth-promoting effects on tall fescue in vitro, as well as on soil-grown tomatoes, and lettuce in various hydroponic systems [16,27]. The present study corroborates these findings, showing increased biomass in IALR632-treated hydroponic lettuce compared to untreated control. The transcriptomic analysis in this study revealed elevated expression of genes associated with phosphorus uptake in lettuce treated with IALR632 (Supplementary Table S6), consistent with prior demonstrations that plant-associated phosphate solubilizing endophytes enhance insoluble phosphate acquisition and promote plant growth [27,28,29]. Additionally, IALR632 led to transcriptional modulation of ethylene biosynthesis, specifically affecting ACO genes, with eight transcripts down-regulated and four up-regulated at 15 DAT (Supplementary Table S6). This was accompanied by significant down-regulation of the GO term response to ethylene (GO:0009723; FDR = 0.02) (Supplementary Table S5), suggesting that IALR632 may attenuate ethylene-mediated stress signaling—a mechanism commonly associated with enhanced plant growth and delayed senescence under non-stress conditions [30,31].

Enrichment of plant growth and development related BP GO terms such as mRNA transcription, lignin catabolic process, response to light stimulus, etc. in IALR632-treated lettuce at 10 DAT suggests that the endophyte exerts a growth-promoting influence starting in the early vegetative phase (Supplementary Table S4). Notably, at 15 DAT, although specific hormone-responsive GO terms such as those linked to auxin (GO:0009733), abscisic acid (GO:0009737), and gibberellin (GO:0009739) were down-regulated, the broader category response to hormone (GO:0009725) showed increased expression (Supplementary Table S5). This suggests a shift toward alternative hormonal signaling pathways, potentially involving cytokinin, salicylic acid, or jasmonic acid [31,32]. The concurrent up-regulation of stress-responsive GO terms, including response to water deprivation and cellular response to heat, may reflect a priming effect, whereby IALR632 enhances basal defense readiness without triggering full stress responses [33]. In hydroponic systems, crops are subjected to osmotic and ionic stress, resulting in reduced water uptake, disrupted nutrient homeostasis, and yield loss [34]. The presence of IALR632 may have mitigated these effects by specifically up-regulating response to water deprivation (GO:0009414). These findings align with previous reports of endophyte-mediated modulation of plant hormone networks to promote resilience and growth under fluctuating environmental conditions.

The presence of a consistent set of highly frequent GO terms at both 10 and 15 DAT suggests that IALR 632 establishes a stable, long-term physiological reprogramming of the lettuce host within the NFT system. The high frequency of auxin-activated signaling (GO:0009734) and cell wall organization (GO:0071555) suggests that the endophyte actively stimulates host growth pathways [35]. This growth is supported by enhanced carbohydrate metabolism (GO:0005975), providing the necessary energy and carbon skeletons. Furthermore, the prominence of defense response (GO:0006952) terms indicates that the endophyte may induce a state of “priming”, enhancing the plant’s resilience to environmental stressors without the growth trade-offs associated with full pathogen activation [36,37]. The observation of regulation of transcription, protein phosphorylation, proteolysis, and transmembrane transport in both up- and down-regulated groups is a key finding. This suggests sophisticated “transcriptomic pruning” or selective reprogramming. Rather than a blanket increase in activity, the plant appears to be selectively up-regulating specific transporters and signaling proteins while simultaneously down-regulating others that may be inhibitory or non-essential for the endophyte-stimulated state. For instance, selective proteolysis and phosphorylation allow the plant to rapidly turn over specific regulatory proteins to maintain an optimized physiological balance for sustained growth [34,38].

In a controlled trial, soilless lettuce plants inoculated with a consortium of plant growth-promoting rhizobacteria (PGPR) were investigated for plant growth, ecophysiology, and metabolic profile under a low-nutrient regime [34]. Investigators found on average the plant biomass increased by 25% in the PGPR-inoculated plants due to enhanced leaf and root growth. PGPR inoculation induced significant metabolic reprogramming in the leaves, affecting several pathways related to growth, development, and stress responses. The results of our lettuce study broadly concur with that study.

Inoculation with *Pseudomonas psychrotolerans* IALR632 led to substantial restructuring of the root-associated bacterial community in hydroponically grown lettuce. At the phylum level, eight of the 18 detected phyla exhibited significant relative abundance changes. Chlamydiae was the only phylum with reduced relative abundance (−50%) in the treated group, which may decrease the prevalence of obligate intracellular bacteria that can divert host resources. In contrast, seven phyla, including Chloroflexi, WPS-2, Planctomycetes, TM7, TM6, and Actinobacteria, were enriched by 34–224% compared to controls. Many of these phyla include members involved in carbon and nitrogen turnover, organic matter decomposition, and production of bioactive metabolites that can benefit plant growth and health.

At the genus level, a total of 83 genera were detected, with the top-ranked group being unclassified taxa that dominated both treatments (~85–87% relative abundance). Among the remaining top 30 most abundant genera, *Methylotenera*, *Rhizobium*, *Methylibium*, and *Sphingomonas* exhibited significantly decreased relative abundance following bacterial inoculation. The decline in these genera may reduce nitrogen loss via denitrification (e.g., *Methylotenera*) or minimize potential competition for root exudates (e.g., *Sphingomonas*). In contrast, several genera were markedly enriched: *Sediminibacterium*, *Hyphomicrobium*, *Sphingobium*, *Devosia*, *Mycobacterium*, *Rhodoplanes*, and *Runella*. These taxa include species reported to participate in degradation of complex organics, mobilization of phosphorus and other nutrients, production of phytohormones, and modulation of rhizosphere signaling pathways. The substantial increase in *Runella*, in particular, may indicate an important role for Bacteroidota-affiliated heterotrophs in recycling organic compounds released by plant roots or microbial activity.

The interesting phenomenon is that *Pseudomonas* genus is not among top 30 most abundant genera. IALR632 inoculation had only 0.045% of genus *Pseudomonas* in relative abundance, lower than 0.065% in control group. Similarly, Yacoub et al. (2024) showed that the abundance level of *Trichoderma* species was low in the samples treated with *Trichoderma* compared with the control samples [39]. This interesting finding implies inoculated endophytes may function through modulating other microbes in complex ways.

Overall, these bacterial compositional changes indicate that IALR632 inoculation promotes a shift toward a more functionally diverse and metabolically active root bacterial microbiome, with greater potential for nutrient mobilization, stress mitigation, and suppression of less beneficial or resource-competing taxa. This restructuring likely contributes to the enhanced growth performance observed in inoculated lettuce under NFT hydroponic conditions.

## 4. Materials and Methods

### 4.1. Lettuce Seeds and Germination

Seed-coated lettuce (*Lactuca sativa* cv. Salanova Green Oakleaf) was purchased from Johnny’s Selected Seeds (Fairfield, ME, USA) and sowed in Oasis Grower Solutions Horticubes XL Foam Medium (276 cells). The trays were placed in AmHydro propagation systems (Arcata, CA, USA) and irrigated with water under natural greenhouse light until germination. After emergence, seedlings were irrigated four times daily (1 min each) with Virginia Tech fertilizer solution [16] at an electrical conductivity (EC) of 1.0 ± 0.1 mS/cm and pH 5.9 ± 0.1, measured using an Economy pH/EC Meter (Spectrum Technologies, Inc., Aurora, IL, USA) and adjusted as necessary. Seedlings were prepared for bacterial inoculation approximately one week after sowing.

### 4.2. Bacterial Strain and Culture

*Pseudomonas psychrotolerans* IALR632 was originally isolated from leaves of *Sorghum halepense* in Central Virginia, USA (37.125372, −79.298415), and its 16S rRNA sequence was deposited in GenBank (accession no. MZ519967) [27]. For inoculation, a loopful from a glycerol stock was grown overnight in 4 mL half-strength Lennox broth (LB) at 30 °C, 200 rpm. This culture was diluted 20-fold in fresh half-strength LB and incubated under the same conditions for ~5 h to an OD_600_ ≈ 1.0 (1.85 × 10^9^ CFU/mL).

### 4.3. Bacterial Inoculation and Plant Transplant

One mL of bacterial suspension above was applied directly to the root zone of each seedling in the propagation system. Control plants received 1 mL of sterile half-strength LB. After one week, seedlings at the 3–4 true leaf stage were transplanted to nutrient film technique (NFT) units described in the following section.

### 4.4. Greenhouse NFT System and Growth Measurements

NFT systems (Model NFT0406, CropKing, Lodi, OH, USA) were used, each with six channels (36-plant capacity) and a slope of 2.5%. Dimensions were 139.7 × 139.7 × 78.74 cm (Supplementary Figure S5). Growth parameters, including shoot and root fresh and dry weights and SPAD readings, were recorded at 14 and 21 days after transplanting.

### 4.5. RNA Sampling and Extraction

Lettuce heads were sampled at 4, 10, and 15 days after transplant (DAT), with four biological replicates per treatment. On each date, four random heads per treatment were harvested, immediately placed in plastic containers, flash-frozen in liquid nitrogen, and stored at −80 °C until RNA extraction. Total RNA was isolated using the Thermo Scientific™ GeneJet Plant RNA Purification Kit (Fisher Scientific, Pittsburgh, PA, USA) following the manufacturer’s instructions, diluted to 100 ng/µL, and shipped to Azenta Life Sciences (South Plainfield, NJ, USA) for RNA-seq.

### 4.6. RNA Sequencing and Transcriptome Analysis

Libraries were constructed and sequenced on an Illumina HiSeq 2000 platform (Illumina, Inc., San Diego, CA, USA) to generate 150 bp paired-end reads. FASTqc v0.12.1, a quality control tool for high throughput data was used to visualize the raw RNA-seq data (http://www.bioinformatics.babraham.ac.uk/projects/fastqc/ (accessed on 10 January 2024)). Since quality of the reads was acceptable, they were uploaded to CyVerse online platform (www.cyverse.org) [40,41] for analysis. Read mapping and transcript assembly (RMTA) app v2.6.3 [42] on CyVerse Discovery environment (DE) was used for the raw read analysis. RMTA is a high throughput RNA-seq read mapping and transcript assembly workflow. CyVerse DE is a web-based graphical user interface for data analysis, visualization, and data sharing. The Genome-Guided Mapping route in RMTA was used to produce a read count table for all the samples. The L. sativa cv. Salinas v11 reference genome and related annotated file in GTF format were downloaded from the genome database of National Library of Medicine (https://www.ncbi.nlm.nih.gov/datasets/genome/GCF_002870075.4/ (accessed on 2 February 2024)) and used for the analysis. Hisat2 (as implemented in the RMTA workflow on CyVerse) [43] option in RMTA was used for aligning reads to the genome. RMTA parameters were configured to target transcripts, with the attribute set to transcript_id for program execution. With this setup, RMTA would identify reads mapped to transcript sequences of the reference genome and provide a count table of reads and their relevant transcript IDs from the annotation file.

The transcripts with low read counts were removed from the table of read counts for downstream analysis. For this, the rows having a sum of read counts < 10 across all replicates related to a comparison (i.e., 4, 10, and 15 DAT) were removed from the table. The filtered data table was used in the Bioconductor package DESeq2 v1.44.0 [44] in R (via RStudio version 2023.12.1+402)to derive differentially expressed transcripts (DETs) between the IALR632 treated and non-treated control samples for sampling days 4, 10, and 15. DETs with adjusted probability (*padj*) < 0.01 were extracted from the DESeq output to a different file. The annotated file of the reference genome was imported into the Bioconductor-rtracklayer package v1.60.1 (https://bioconductor.org/packages/3.18/bioc/html/rtracklayer.html (accessed on 2 February 2024)) of the R program to easily look up and extract genomic features and attributes. The gene ID and gene description corresponding to each DET were also extracted and merged with the output file of DESeq2 analysis. The corresponding gene IDs of DETs (*padj* < 0.01) were checked for duplicates to see if all gene IDs were unique or if there were any genes corresponding to two or more transcript IDs.

### 4.7. MapMan Analysis

Pathway analysis of lettuce samples collected at different time points was carried out using the MapMan tool version 3.7.0 [45]. For this purpose, a MapMan mapping file was generated specifically for lettuce genes through the Mercator platform (https://www.plabipd.de/mercator_main.html (accessed on 13 November 2025)). In this process, genes were assigned into functional bins by Mercator4, which organizes them according to hierarchical ontologies.

### 4.8. DAVID Analysis

The web-based bioinformatics tool “Database for Annotation, Visualization, and Integrated Discovery” (DAVID) (https://davidbioinformatics.nih.gov/) [46,47] was used to understand the biological meaning of differentially expressed transcripts in each comparison. The DAVID tool was used to identify enriched biological themes and Gene Ontology (GO) terms and discover functional annotations of enriched gene groups. For each comparison, the original read counts table was examined to identify genes with no detectable expression across any of the samples. These non-expressed genes were excluded, and the resulting filtered gene list was used to construct the reference genome for DAVID analysis. It is necessary to remove unexpressed genes from the reference genome in order to avoid false positives and accurately calculate gene enrichment of differentially expressed genes (DEGs). The DEGs were sorted according to log2fold values, and up- and down-regulated genes were analyzed for each comparison separately using DAVID’s default parameters. Biological process (BP) terms from DAVID’s GOTERM_Direct database were examined to elucidate functional patterns in the tested samples. The online multiple list comparator tool hosted by https://molbiotools.com/ was used to prepare Venn diagrams for up- and down-regulated DEGs separately to understand the number of unique and common genes in each comparison.

### 4.9. Root Sampling and DNA Isolation

Root samples were taken at 14 DAT with 4 biological replicates and immediately placed in liquid nitrogen and stored at −80 °C until DNA extraction. Genomic DNA was isolated from root samples using NucleoSpin Plant II, Mini kit for DNA from plants (Macherey-Nagel, Allentown, PA, USA) following the manufacturer’s instructions. The genomic DNA was quantified with Quant-iT™ PicoGreen™ dsDNA Assay (Invitrogen, Carksbad, CA, USA). The DNA solutions were diluted to 20 ng/µL and shipped to Azenta Life Sciences (South Plainfield, NJ, USA) for bacterial microbiome sequencing.

### 4.10. Library Construction and Miseq Sequencing

The three variable regions of 16S rDNA (V3, V4, V5) were amplified and identified various species including archaea. Illumina MiSeq sequencing platform was used for 16S rDNA amplicon sequencing and pair-end chemistry enabled to read as long as 600 bp.

### 4.11. Bioinformatics Analysis Workflow

Sequencing data quality optimization was conducted using the software packages Cutadapt (v1.9.1), Vsearch (1.9.6), and Qiime (1.9.1). First, the two sequences of each read pair were merged according to overlapping sequences with at least 20 bp long. Then, primer and adapter sequences were removed. The 5′ and 3′ bases with Q scores lower than 20 were removed. The resulting sequences with length > 200 bp would pass this step of processing. Finally, the sequences obtained were then aligned to UCHIME ‘Gold’ database to identify and remove chimera sequences. Sequences that passed this filtering step were deemed as clean data ready for analysis.

### 4.12. OTU Analysis and Species Annotation of Root Bacterial Microbiome

OTU (Operational Taxonomic Unit) clusters were defined by a 97% identity threshold for data statistics and analysis using the software packages Qiime (1.9.1) and Vsearch (1.9.6). Unique sequences were extracted from the optimized sequences with the read count information, and all optimized sequences were compared with OTU representative sequences. The sequences with >97% similarity to a specific OTU representative sequence were assigned to the same OTU. To obtain the classification information of OTUs, a representative sequence was selected for each OTU and annotated using the RDP classifier Bayesian algorithm, thereby obtaining the community composition of each sample.

## 5. Conclusions

This study demonstrates that *Pseudomonas psychrotolerans* IALR632 markedly enhances lettuce growth under hydroponic conditions by driving substantial transcriptomic shifts in pathways associated with cell wall remodeling, lipid metabolism, nitrogen assimilation, and hormone signaling. In parallel, the root bacterial microbiome was restructured toward taxa linked to improved nutrient mobilization and stress resilience, as supported by robust diversity metrics and ordination analyses. Future work should incorporate comprehensive characterization of additional microbiome components—particularly fungal communities, including potential fungal pathogens—to obtain a more complete understanding of plant–microbe interactions in hydroponic systems. Moreover, exploring microbial consortia that combine IALR632 with other beneficial microorganisms will be essential for developing effective, scalable, and practical biostimulant strategies for commercial hydroponic production.

## Data Availability

The data presented in this study are openly available in the NCBI Sequence Read Archive at https://www.ncbi.nlm.nih.gov/sra/PRJNA1348882 (accessed on 26 October 2025), reference number PRJNA1348882. Some data supporting the findings of this study are included in the Supplementary Information Files. Any other data are available from the corresponding author upon reasonable request.

## Supplementary Materials

The following supporting information can be downloaded at: https://www.mdpi.com/article/10.3390/ijms27073072/s1.

## Abbreviations

The following abbreviations are used in this manuscript:

ACC	1-aminocyclopropane-1-carboxylic acid
ANOSIM	Analysis of similarities
BP	Biological process
CEA	Controlled environment agriculture
CFU	Colony forming unit
DAT	Days after transplanting
DAVID	Database for Annotation, Visualization, and Integrated Discovery
DEG	Differentially expressed genes
DET	Differentially expressed transcript
DW	Dry weight
ERF	Ethylene-responsive transcription factor
FW	Fresh weight
GO	Gene ontology
LB	Lennox broth
NFT	Nutrient film technique
NMDS	Non-metric multidimensional scaling
OD	Optical density
OPP	Oxidative pentose phosphate
OTU	Operational taxonomic unit
PCoA	Principal Coordinates Analysis
PGPB	Plant growth-promoting bacteria
PGPR	PGPR: Plant growth-promoting rhizobacteria
RMTA	Read mapping and transcript assembly
SPAD	Soil plant analysis development
TCA	Tricarboxylic acid cycle
