# Supplementary material for: Transcriptomic and Root Microbiome Responses of Lettuce to Beneficial Endophytic Bacteria in Hydroponic Systems
Source: Int J Mol Sci. 2026 Mar 27;27(7):3072. doi: 10.3390/ijms27073072 (PMC13074086; doi:10.3390/ijms27073072)
Supplement: Supplementary file 1 [file ijms-27-03072-s001.zip › ijms-4199728-supplementary/Suppl table and figures.pdf]

Supplementary Table S1. Overview of the lettuce transcriptomic data set.

Treatment*	Average total number of RNA-seq reads (millions)	Average % of aligned reads to the reference genome	Average % of assigned reads to annotated features of the reference genome
CK-4d	38.10	92.44	80.16
T-4d	36.10	91.49	79.42
CK-10d	38.68	91.55	77.62
T-10d	37.50	91.45	78.20
CK-15d	38.05	91.52	78.53
T-15d	43.79	88.41	76.64

*CK: Control; T: Bacterial treatment; 4d, 10d, and 15d: the sampling points depicted as number of days after transplanting. Lettuce cv. Salinas v11 was used as reference genome. Lettuce samples were taken 4, 10, and 15 days after transplanting for transcriptome profiling with four biological replicates.

Supplementary Table S2. Output from DESeq2 differential expression analysis of lettuce (*Lactuca sativa* cv. Green Oakleaf) 11, 17, and 22 days after treated with *Pseudomonas psychrotolerans* strain IALR 632, relative to untreated control. Differentially expressed transcripts were identified using an adjusted p-value threshold of Padj ≤ 0.01. This table also incorporates transcript count data generated via the Read Mapping and Transcript Assembly (RMTA) pipeline. Each transcript is annotated with its corresponding gene ID and associated metadata from the *L. sativa* cv. Salinas v11 reference genome.

Supplementary Table S3. Enriched Biological Process GO Terms (GOTERM_BP_DIRECT) at 4 days after transplanting in lettuce (*Lactuca sativa*) cv. Green Oakleaf plants. The plants had been inoculated with the bacterial endophyte *Pseudomonas psychrotolerans* strain IALR 632, and differentially expressed genes were extracted at Padj = 0.01 in comparison to untreated control. There were no enriched GO terms for up- regulated genes.

Supplementary Table S4. Enriched Biological Process GO Terms (GOTERM_BP_DIRECT) associated with differentially expressed genes of lettuce (*Lactuca sativa*) cv. Green Oakleaf plants at 10 days post transplanting. The plants were inoculated with the bacterial endophyte *Pseudomonas psychrotolerans* strain IALR 632, and differentially expressed genes were extracted at Padj = 0.01 in comparison to untreated control.

Supplementary Table S5. Enriched Biological Process GO Terms (GOTERM_BP_DIRECT) associated with differentially expressed genes of lettuce (Lactuca sativa) cv. Green Oakleaf plants 15 days post transplanting. The plants were inoculated with the bacterial endophyte *Pseudomonas psychrotolerans* strain IALR 632, and differentially expressed genes were extracted at Padj = 0.01 in comparison to untreated control.

Supplementary Table S6. Common Gene Ontology (GO) terms with relatively high frequency in Lactuca sativa cv. Green Oakleaf transcriptomes sampled at 10 and 15 days post-transplanting. Plants were inoculated with the endophytic bacterium *Pseudomonas psychrotolerans* strain IALR 632, and differentially expressed genes were identified at Padj = 0.01 relative to untreated controls.

GO Term Description	Gene Regulation	No. of genes at 10 DAT*	No. of genes at 15 DAT
GO:0006952~defense response	Up	20	87
GO:0006355~regulation of DNA-templated transcription	Up	13	138
GO:0006508~proteolysis	Up	12	53
GO:0055085~transmembrane transport	Up	9	56
GO:0005975~carbohydrate metabolic process	Up	8	56
GO:0006468~protein phosphorylation	Up	7	147
GO:0032259~methylation	Up	6	45
GO:0071555~cell wall organization	Up	6	40
GO:0009734~auxin-activated signaling pathway	Up	5	20
GO:0006355~regulation of DNA-templated transcription	Down	25	136
GO:0006468~protein phosphorylation	Down	24	175
GO:0055085~transmembrane transport	Down	17	125
GO:0006508~proteolysis	Down	12	74
GO:0006633~fatty acid biosynthetic process	Down	6	24

*DAT: days after transplanting

Supplementary Table S7. Differential expression of phosphate solubilization and ethylene biosynthesis-related genes in lettuce (*Lactuca sativa* cv. Green Oakleaf) plants treated with endophyte IALR632, compared to untreated controls. Genes exhibiting upregulation or downregulation at 10 and 15 days post-transplantation (DAT) are listed.

Gene function	DAT	Regulation	Transcript ID	Gene ID	Log2 fold	Product
Phosphate solubilizing	10	Up	XM_023894171.3	LOC111898238	2.12	acid phosphatase 1
			XM_023900181.3	LOC111904408	0.66	purple acid phosphatase 8
	15	Up	XM_023894171.3	LOC111898238	3.05	acid phosphatase 1
			XM_023900411.3	LOC111904674	1.90	acid phosphatase 1
			XM_023913951.3	LOC111918260	1.23	purple acid phosphatase
			XM_023911559.3	LOC111915916	0.43	purple acid phosphatase 23
		Down	XM_023878198.2	LOC111881805	-0.46	bifunctional purple acid phosphatase 26
			XM_023916725.3	LOC111921153	-0.54	purple acid phosphatase 7

			XM_023900536.2	LOC111904822	-1.00	purple acid phosphatase 22
Ethylene-responsive transcription factors	10	Up	XM_023875898.3	LOC111879435	0.63	AP2-like ethylene-responsive transcription factor At2g41710
			XM_023902687.3	LOC111906900	0.35	ethylene-responsive transcription factor RAP2-12
		Down	XM_023916846.3	LOC111921270	-0.42	ethylene-responsive transcription factor RAP2-4
			XM_023906209.3	LOC111910365	-0.63	ethylene-responsive transcription factor RAP2-12
			XM_023899006.3	LOC111903232	-0.69	ethylene-responsive transcription factor ERF027
			XM_023907652.3	LOC111911902	-0.70	ethylene-responsive transcription factor WIN1
	15	Up	XM_023906686.3	LOC111910881	3.04	AP2-like ethylene-responsive transcription factor AIL5
			XM_023898907.3	LOC111903119	2.87	AP2-like ethylene-responsive transcription factor ANT
			XM_023913741.3	LOC111918086	2.49	ethylene-responsive transcription factor ERF014
			XM_023891071.3	LOC111894983	2.45	ethylene-responsive transcription factor 12
			XM_023906518.3	LOC111910687	2.40	AP2-like ethylene-responsive transcription factor ANT
			XM_023879798.3	LOC111883473	2.39	AP2-like ethylene-responsive transcription factor AIL6
			XM_052766817.1	LOC111889043	1.84	ethylene-responsive transcription factor ERF071
			XM_052767383.1	LOC111903849	1.61	AP2-like ethylene-responsive

						transcription factor AIL1
			XM_023885038.3	LOC111888900	1.58	ethylene-responsive transcription factor CRF4
			XM_023911236.3	LOC111915587	1.42	AP2-like ethylene-responsive transcription factor AIL6
			XM_023909673.1	LOC111913959	1.39	ethylene-responsive transcription factor 2
			XM_023906743.1	LOC111910972	1.38	ethylene-responsive transcription factor CRF6
			XM_023880229.3	LOC111883899	1.32	ethylene-responsive transcription factor TINY
			XM_023908059.3	LOC111912333	1.21	ethylene-responsive transcription factor ERF113
			XM_023890547.3	LOC111894473	1.19	ethylene-responsive transcription factor ERF118
			XM_023904337.3	LOC111908515	0.96	ethylene-response factor C3
			XM_023901754.3	LOC111906015	0.86	ethylene-responsive transcription factor ERF038
			XM_023875898.3	LOC111879435	0.72	AP2-like ethylene-responsive transcription factor At2g41710
			XM_023900302.3	LOC111904549	0.49	ethylene-responsive transcription factor ERF118
		Down	XM_023893286.3	LOC111897329	-0.23	ethylene-responsive transcription factor TINY
			XM_023900655.2	LOC111904967	-0.29	ethylene-responsive transcription factor 3
			XM_023888871.3	LOC111892828	-0.52	ethylene-responsive transcription factor 3
			XM_023907386.3	LOC111911628	-0.61	ethylene-responsive transcription factor ERF011
			XM_023890371.3	LOC111894293	-0.62	ethylene-responsive transcription factor ERF061

			XM_023907652.3	LOC111911902	-0.66	ethylene-responsive transcription factor WIN1
			XM_023905992.3	LOC111910207	-0.67	ethylene-responsive transcription factor WIN1
			XM_023916846.3	LOC111921270	-0.70	ethylene-responsive transcription factor RAP2-4
			XM_023872695.3	LOC111876171	-0.72	ethylene-responsive transcription factor ERF010
			XM_023902492.3	LOC111906718	-0.77	ethylene-responsive transcription factor 4
			XM_023908391.3	LOC111912637	-0.80	ethylene-responsive transcription factor ERF010
			XM_023912953.3	LOC111917268	-0.84	ethylene-responsive transcription factor RAP2-3
			XM_052766082.1	LOC111897076	-0.90	ethylene-responsive transcription factor RAP2-7, transcript variant X1
			XM_023914415.3	LOC111918789	-1.23	ethylene-responsive transcription factor ERF061
			XM_023898547.3	LOC111902738	-1.24	ethylene-responsive transcription factor ERF011
			XM_023911854.2	LOC111916239	-1.83	ethylene-responsive transcription factor ERF025
			XM_023899006.3	LOC111903232	-1.92	ethylene-responsive transcription factor ERF027
ACC oxidase (ACO)	10	Up	XM_023901440.3	LOC111905714	5.73	1-aminocyclopropane-1-carboxylate oxidase 1
			XM_023884046.3	LOC111887911	2.17	1-aminocyclopropane-1-carboxylate oxidase homolog 1
			XM_052770096.1	LOC111915034	0.91	-do-
		Down	XM_023906824.3	LOC111911042	-0.56	-do-
			XM_023911476.3	LOC111915839	-0.59	-do-
			XM_023884193.3	LOC111888087	-0.87	-do-
			XM_023884183.3	LOC111888080	-1.00	-do-
	15	Up	XM_023901440.3	LOC111905714	5.6	1-aminocyclopropane-1-carboxylate oxidase 1

			XM_023884046.3	LOC111887911	3.46	1-aminocyclopropane-1-carboxylate oxidase homolog 1
			XM_023884211.3	LOC111888100	3.11	-do-
			XM_052770096.1	LOC111915034	1.01	-do-
		Down	XM_023884195.3	LOC111888088	-0.46	-do-
			XM_023887049.2	LOC111890969	-0.68	1-aminocyclopropane-1-carboxylate oxidase 3
			XM_023893213.3	LOC111897251	-0.79	1-aminocyclopropane-1-carboxylate oxidase homolog 1
			XM_023884198.3	LOC111888091	-0.94	-do-
			XM_023906824.3	LOC111911042	-0.99	-do-
			XM_023911476.3	LOC111915839	-1.23	-do-
			XM_023884183.3	LOC111888080	-1.62	-do-
			XM_023884193.3	LOC111888087	-1.74	-do-

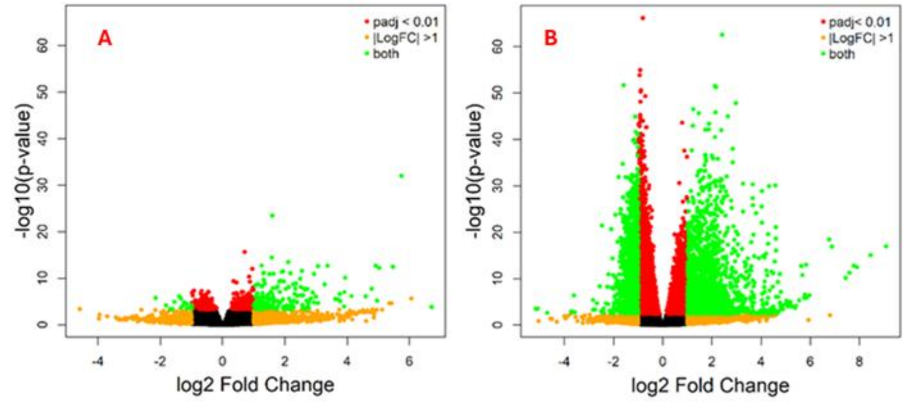

Fig. S1. Volcano plots of differentially expressed genes in lettuce (Lactuca sativa cv. Green Oakleaf) at 10 and 15 days after transplanting (DAT). Lettuce seedlings were inoculated with endophyte IALR632 and compared to none-inoculated seedlings for gene expression. A. At 10 DAT, a total of 17,614 genes were expressed, of which 796 had adjusted p values < 0.01 (red dots) and 1,900 showed |log2 fold change| > 1 (orange dots). Among these, 210 genes satisfied both thresholds (green dots). B. At 15 DAT there were 20722 expressed genes, and the corresponding numbers were 7,642, 4,023, and 2,342, respectively. The p values and log2 fold changes for all expressed genes are provided in Supplementary Table 2.

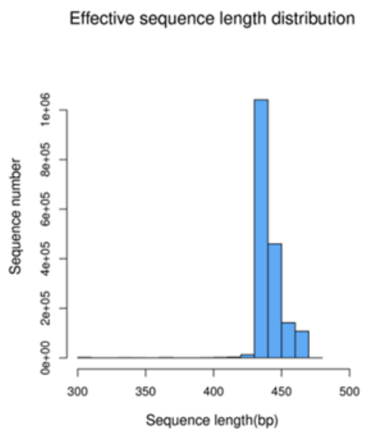

Fig. S2. The distribution of effective sequence lengths.

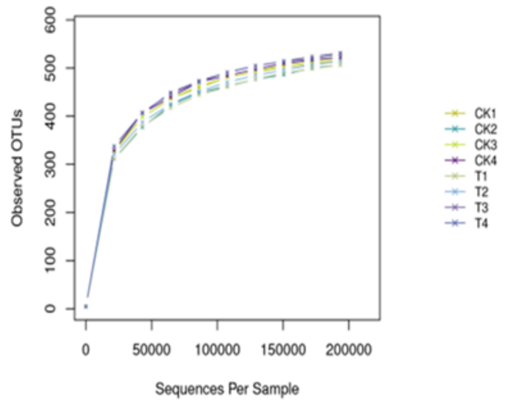

Fig. S3. The rarefaction curves of sequences per sample.

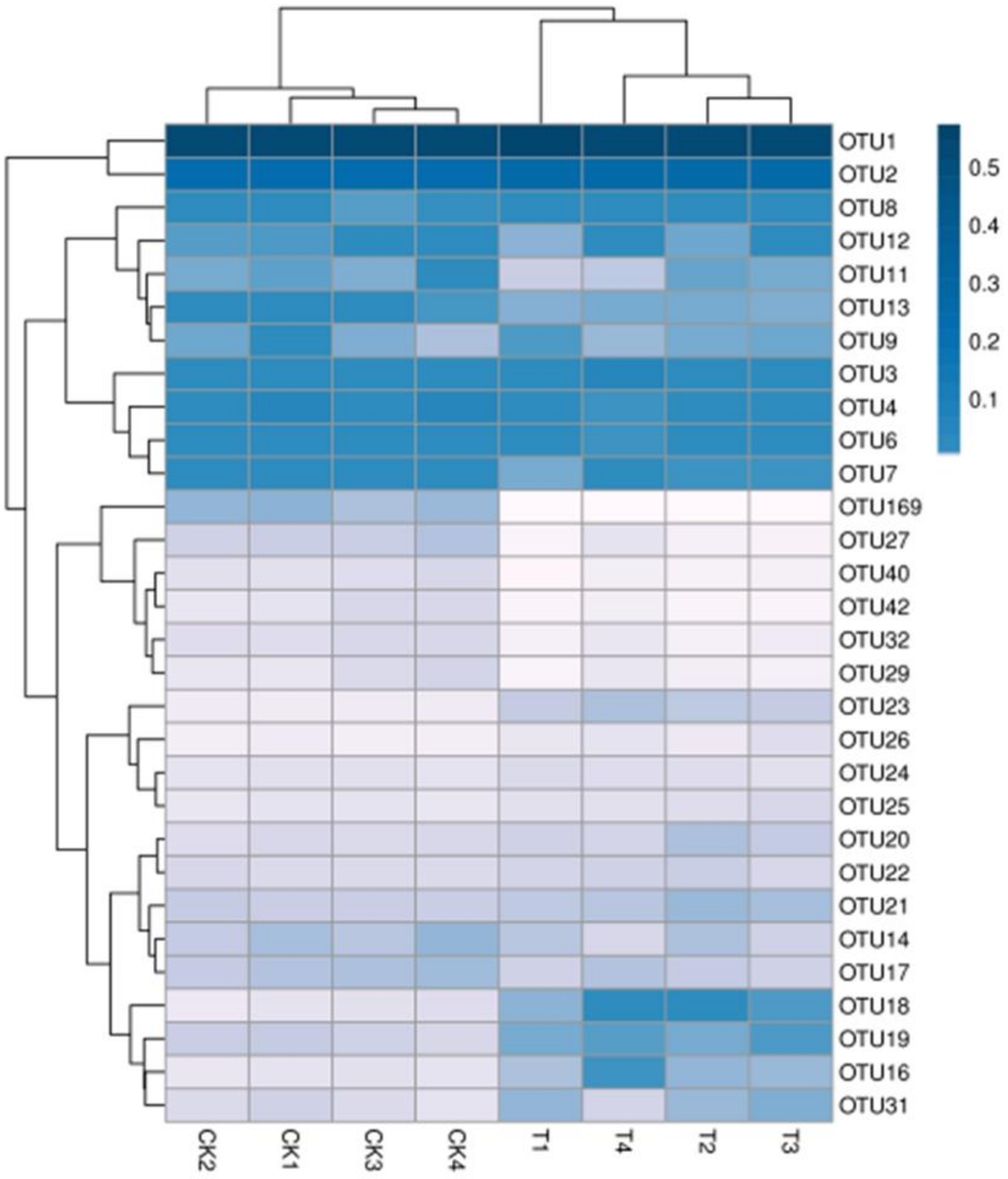

Fig. 4S. Heatmap of the top 30 most abundant OTUs across control and IALR632 treatment groups.

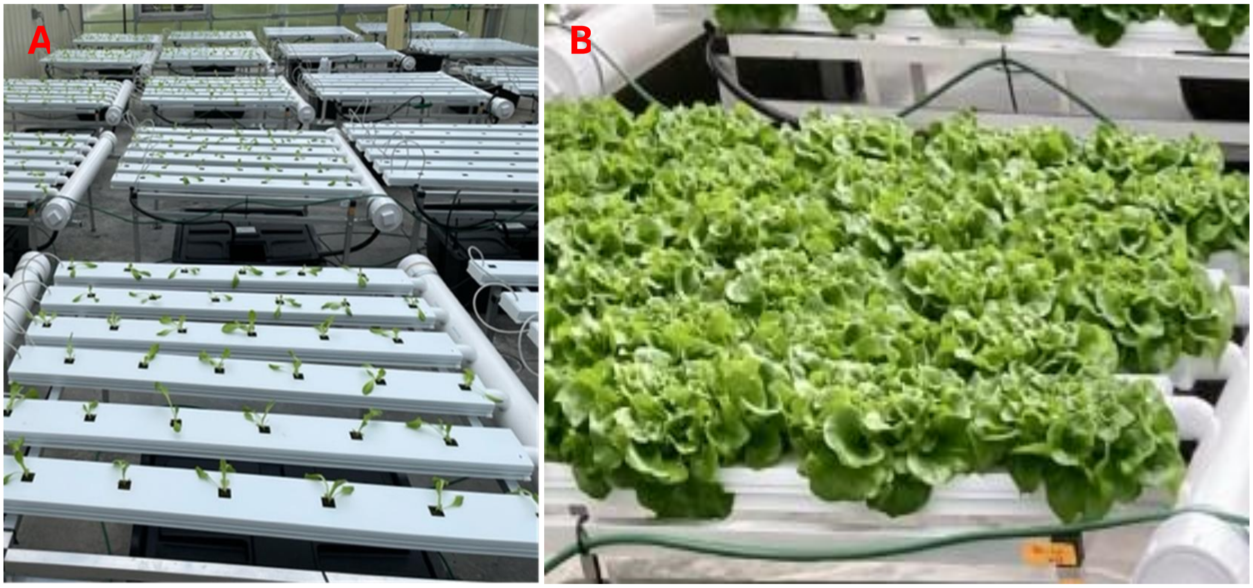

Fig. 5S. Pictures of nutrient film technique (NFT) units with lettuce plants grown in greenhouse. A. Lettuce plants just transplanted. B. Lettuce plants grown in NFT units for 3 weeks.
